# A Case of Non-obstetric Ovarian Vein Thrombosis Associated With Acute Pyelonephritis Presenting to the Emergency Department

**DOI:** 10.7759/cureus.106306

**Published:** 2026-04-01

**Authors:** Hamza Yousfi, Nada Essarraje, Benoit Vokaer, Adeline Higuet, Frederic Vandergheynst

**Affiliations:** 1 Department of Emergency Medicine, Hôpital Universitaire de Bruxelles (HUB), Erasme Hospital, Université Libre de Bruxelles (ULB), Brussels, BEL; 2 Department of Internal Medicine, Hôpital Universitaire de Bruxelles (HUB), Erasme Hospital, Université Libre de Bruxelles (ULB), Brussels, BEL

**Keywords:** computed tomography, emergency department, ovarian vein thrombosis, pyelonephritis, venous thromboembolism

## Abstract

Ovarian vein thrombosis (OVT) is a rare type of venous thromboembolism (VTE) that is most often reported during the postpartum period and is rarely seen outside pregnancy. In non-obstetric settings, its presentation is often nonspecific and may resemble more common urinary, gastrointestinal, or gynecologic emergencies. It may also be missed on contrast-enhanced abdominopelvic CT, particularly when CT interpretation is biased toward a more common diagnosis.

We report the case of a 36-year-old woman who presented to the emergency department (ED) with sudden-onset abdominal and pelvic pain, urinary symptoms, and significant systemic inflammation. Initial contrast-enhanced CT was interpreted as left-sided acute pyelonephritis, and antibiotic therapy was started. On day three, a subsequent review of the same CT examination by an attending radiologist identified an extensive left-sided OVT that had been missed on the preliminary on-call CT report, and the patient was recalled to the ED for initiation of therapeutic anticoagulation. Rivaroxaban was prescribed for three months, with a good clinical course.

This report highlights non-obstetric OVT as an uncommon but important differential diagnosis in women with acute abdominal or pelvic pain that may closely resemble urinary, gastrointestinal, or gynecologic emergencies. A systematic evaluation of the ovarian and other abdominopelvic veins on contrast-enhanced CT, with additional review by an attending radiologist when appropriate, may help reduce the risk of missed diagnoses.

## Introduction

Ovarian vein thrombosis (OVT) is a rare type of venous thromboembolism (VTE), most commonly reported in the postpartum period, where it occurs in approximately 0.01-0.19% of deliveries [1]. Outside this setting, its incidence remains poorly defined, and it is often not included in the differential diagnosis [1-4]. Its clinical presentation is nonspecific and may resemble more common abdominal and pelvic emergencies in women, particularly urinary, gastrointestinal, and gynecologic conditions [1,4]. Consequently, OVT may be missed on initial interpretation of contrast-enhanced abdominopelvic CT when attention is directed toward a more common and clinically plausible diagnosis. Although non-pregnancy-related OVT has been previously reported, cases occurring in the setting of acute pyelonephritis are uncommon [5-6].

This case report highlights the importance of systematically evaluating the ovarian and other abdominopelvic veins on CT and, when feasible, obtaining a final review from an attending radiologist for preliminary CT interpretations, particularly during on-call hours. We report the case of a 36-year-old woman who presented to the emergency department (ED) with a left-sided OVT in the setting of acute pyelonephritis. The initial CT was interpreted as showing acute pyelonephritis alone, and the OVT was detected only during the final review of the same examination by an attending radiologist.

## Case presentation

A 36-year-old woman presented to the ED on day zero with a history notable for an appendectomy. Her obstetric history was gravida 4, para 2, with two vaginal deliveries and two pregnancy terminations. She reported smoking five cigarettes daily for 15 years and occasional alcohol use. She had no known drug allergies. She was taking a combined oral contraceptive containing ethinyl estradiol/levonorgestrel (Microgynon® 20) and had no personal or family history of VTE.

The patient reported severe, diffuse abdominal pain that began the day before presentation, predominantly involving the left lower quadrant and suprapubic region, with radiation to the back and notable left-sided low back pain. This was associated with chills, subjective fever, vomiting, and increased urinary frequency without dysuria. She had taken 1 g of acetaminophen approximately three hours before presentation and had not received any prior antibiotics. Her last menstrual period was approximately four weeks earlier. She denied any gynecologic symptoms. On examination, diffuse abdominal tenderness was noted, most pronounced in the left lower quadrant, left flank, and suprapubic region, without guarding or rebound. Tenderness at the left costovertebral angle was also present, and the remainder of the examination was unremarkable. Vital signs were as follows: temperature 36.9° C, blood pressure 115/90 mmHg, heart rate 110 beats per minute, and oxygen saturation 100% on room air.

Laboratory and urinary findings on day zero (Table 1) demonstrated significant inflammation, with neutrophilic leukocytosis (white blood cell count (WBC): 20,100/mm³; neutrophils: 15,200/mm³) and a C-reactive protein (CRP) level of 117 mg/L, without evidence of renal impairment. Urinalysis showed leukocyte esterase 2+, pyuria (314 leukocytes/µL), and microscopic hematuria (55 erythrocytes/µL), whereas the urine culture was negative. Urine pregnancy testing was negative both at home and in the ED. CT was performed because of the severity and diffuse distribution of the abdominal pain, the marked inflammatory response, and the broad differential diagnosis, which included gastrointestinal, urinary, and gynecologic causes such as bowel obstruction, gastrointestinal perforation, diverticulitis, upper urinary tract infection (UTI), renal colic, pelvic inflammatory disease, adnexal torsion, and ruptured ovarian cyst.

**Table 1:** Key laboratory, urinary, and pregnancy test results on day zero and day three Reference ranges correspond to local laboratory values CRP: C-reactive protein; —: not performed

Parameter	Day 0	Day 3	Reference range
White blood cell count, n/mm^3^	20,100	9,800	3,500-11,000
Neutrophils, n/mm^3^	15,200	6,280	1,500-6,700
CRP, mg/L	117	57	<5
Serum creatinine, mg/dL	0.80	0.62	0.50-0.90
D-dimer, ng/mL	—	530	<500
Urinalysis - leukocyte esterase	2+	—	
Urinalysis - leukocytes, n/µL	314	—	<20
Urinalysis - erythrocytes, n/µL	55	—	<12
Urine culture	Negative	—	Negative
Urine pregnancy test	Negative	—	Negative

**Figure 1:**
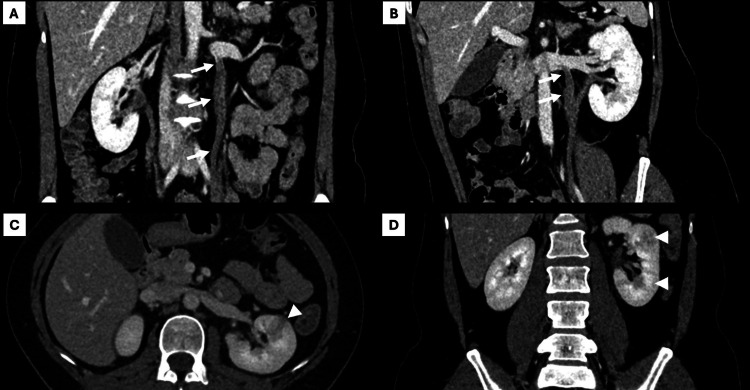
Contrast-enhanced abdominopelvic CT showing left OVT with concurrent ipsilateral acute pyelonephritis (A-B) Coronal multiplanar reconstructions show a dilated left ovarian vein with an intraluminal low-attenuation filling defect, consistent with left OVT (arrows). (C-D) Axial and coronal images show features of acute pyelonephritis, including a striated nephrogram and focal wedge-shaped areas of decreased enhancement in the left kidney (arrowheads) CT: computed tomography; OVT: ovarian vein thrombosis

CT demonstrated an enlarged left kidney with multiple wedge-shaped hypoenhancing areas and diffuse enhancing thickening of the left ureteral wall, findings consistent with left-sided acute pyelonephritis (Figure 1). Taken together, the clinical, laboratory, and imaging findings supported this diagnosis, and empiric antibiotic therapy was initiated according to local guidelines (ciprofloxacin 500 mg orally twice daily for seven days), with clinical reassessment recommended.

On day three, the ED was informed by an attending radiologist that an extensive left OVT, missed on the preliminary on-call CT report, had been identified on the final review of the same CT examination (Figure 1). The patient was therefore recalled the same day, and her management was revised. At reassessment, she reported improvement in abdominal pain and resolution of urinary symptoms. Vital signs were stable. Examination showed persistent left-sided abdominal and costovertebral angle tenderness. Laboratory results on day three, summarized in Table 1, showed improvement in inflammatory markers (WBC: 9,800/mm³; CRP: 57 mg/L), and a D-dimer of 530 ng/mL.

Thrombophilia and antiphospholipid antibody testing, performed before initiation of anticoagulation, were negative. The patient reported mild exertional dyspnea, but there was no hypoxemia, hemodynamic instability, chest pain, or hemoptysis. No YEARS criteria were present, and the D-dimer level was below the 1000 ng/mL threshold [7]. Therefore, CT pulmonary angiography (CTPA) was not performed, as therapeutic anticoagulation for OVT was already indicated. Therapeutic anticoagulation was initiated with rivaroxaban 15 mg twice daily for 21 days, followed by 20 mg once daily to complete a three-month course. Ciprofloxacin was continued. She was discharged with scheduled hematology and gynecology follow-up.

At the six-week hematology follow-up, the patient had mild residual abdominal tenderness without evidence of complications or symptomatic recurrence. Anticoagulation was planned for a total duration of three months, and an abdominal ultrasound was requested to assess ovarian vein patency. Contraceptive counseling included a recommendation to switch to a drospirenone-only pill. At the six-month follow-up, she remained clinically well. An abdominal ultrasound performed at that visit was primarily obtained to reassess the left kidney after the episode of pyelonephritis and showed otherwise unremarkable renal findings, with a normal-appearing left kidney measuring 11.7 cm and no pelvicalyceal dilatation.

Because the examination was not targeted at the ovarian veins, ovarian vein patency could not be determined. A contrast-enhanced abdominopelvic CT scan was scheduled one month later to document thrombus evolution, but it was canceled after an early pregnancy was identified. At the eight-month follow-up, obstetric ultrasound confirmed a viable intrauterine pregnancy at approximately seven weeks’ gestation. Given the patient's recent history of unusual-site VTE, prophylactic enoxaparin 4,000 anti-Xa IU once daily was initiated.

## Discussion

OVT is a rare form of venous thromboembolism (VTE), most commonly described in the postpartum period, complicating approximately 0.01-0.19% of deliveries [1]. Outside pregnancy-related settings, its incidence is poorly defined, and it may not be routinely considered in the initial differential diagnosis [1,4]. OVT shows a marked right-sided predominance [1,3] and is substantially less common than lower-extremity deep vein thrombosis (DVT) [2]. Beyond the obstetric setting, OVT typically occurs in the presence of identifiable predisposing conditions, such as malignancy, recent surgery, estrogen exposure, intra-abdominal infection, or inflammatory disease, whereas idiopathic cases account for only a minority of presentations [1-4]. This report highlights that non-obstetric OVT may be overlooked on contrast-enhanced abdominopelvic CT when concomitant acute pyelonephritis provides a more common and clinically plausible explanation for the presentation. The atypical left-sided location may have further lowered clinical suspicion. Together, these features support the systematic assessment of the ovarian and other abdominopelvic veins on CT in non-pregnant women presenting to the ED with acute abdominal or pelvic pain.

A focused literature search of PubMed/MEDLINE, supplemented by Google Scholar and manual screening of reference lists (from database inception to February 1, 2026), identified two clearly relevant non-obstetric case reports. Bruening et al. described an 81-year-old woman with right-sided OVT and concurrent ipsilateral acute pyelonephritis [5], whereas Pak et al. reported a 46-year-old woman with left-sided OVT associated with acute pyelonephritis and ureteritis, described as “UTI-induced OVT” [6]. In both reports, urine cultures grew *Escherichia coli* [5-6]. Our case findings are broadly consistent with these observations, suggesting that infection may be a possible contributing factor in non-obstetric OVT, but differ in that acute pyelonephritis was not microbiologically confirmed, despite compatible urinary symptoms, elevated inflammatory markers, positive urinalysis, and CT findings suggestive of acute pyelonephritis.

By contrast, most other retrieved case reports concerned the postpartum period, in which OVT may mimic pyelonephritis without an upper UTI being clearly established as the precipitating factor for thrombosis [8,9]. In addition, several reviews and case series list infection-related settings, including pyelonephritis or UTI, among reported associations, but often provide limited individual-level data and do not consistently distinguish pregnancy-related from non-pregnancy-related cases, thereby limiting interpretation of these findings [1,3,10]. Overall, this case adds to the limited literature on non-obstetric OVT occurring in the setting of acute pyelonephritis and, to our knowledge, is among the few reports providing detailed individual clinical, imaging, management, and follow-up data.

**Figure 2:**
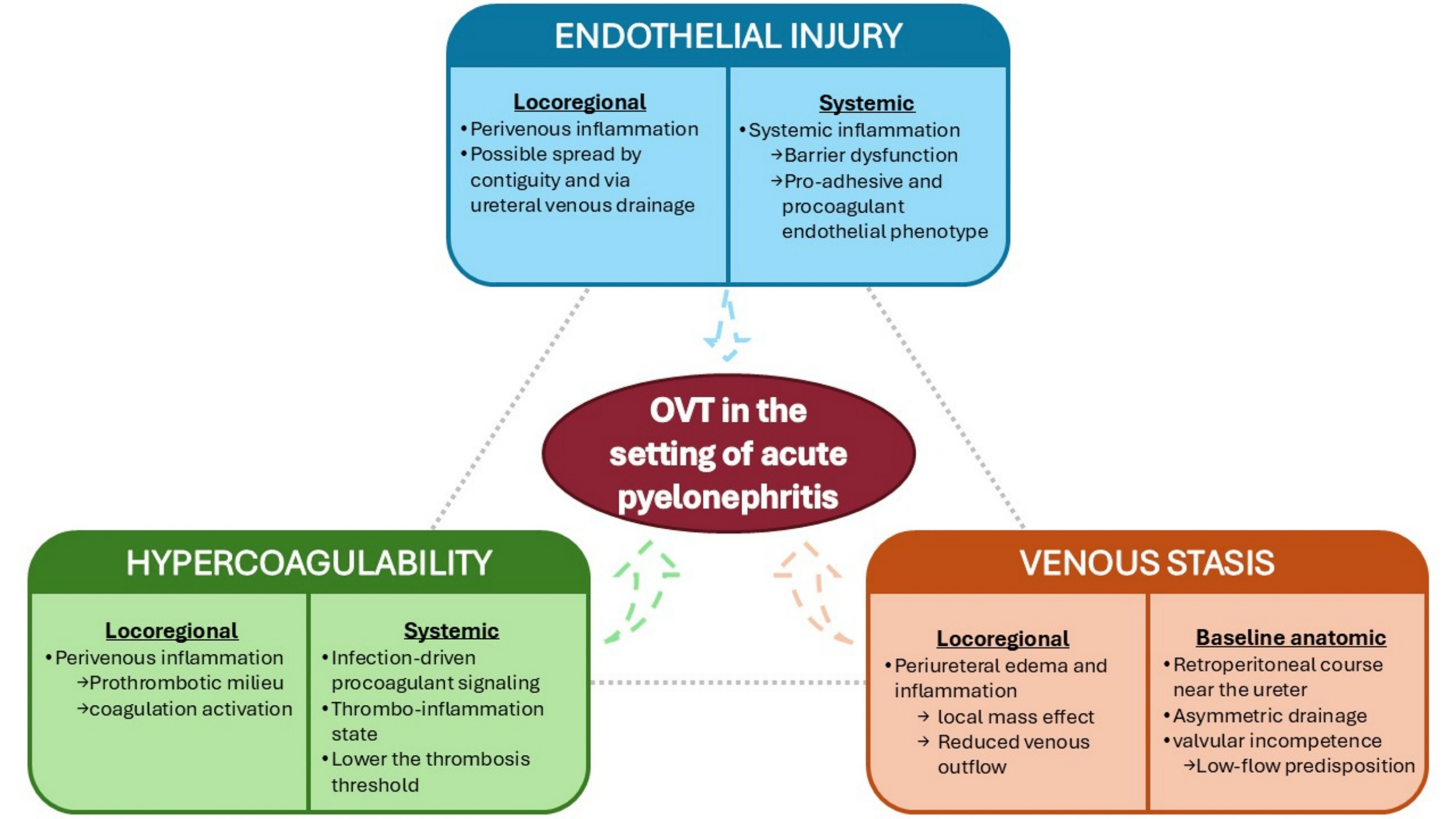
Proposed mechanisms linking acute pyelonephritis to OVT through amplification of Virchow’s triad via locoregional and systemic inflammatory pathways The figure summarizes the hypothesized contributions of venous stasis, endothelial injury, and hypercoagulability in this clinical setting OVT: ovarian vein thrombosis This original schematic figure was created by the first author using Microsoft PowerPoint (Microsoft Corporation, Redmond, WA) and exported as a JPEG image

A limitation of this case report is that acute pyelonephritis was not microbiologically confirmed because the urine culture remained negative. However, urine cultures may remain negative in a substantial proportion of patients with acute pyelonephritis, particularly when the diagnosis is supported by compatible clinical and imaging findings [11,12]. This distinguishes our case from the two previously published non-obstetric reports, both of which had microbiologic documentation of *Escherichia coli* UTI [5-6], but does not exclude acute pyelonephritis in the present patient. In this patient, urinary symptoms, marked inflammatory response, positive urinalysis, and CT abnormalities interpreted as consistent with acute pyelonephritis supported the diagnosis. In non-obstetric OVT occurring in the setting of acute pyelonephritis, Virchow’s triad provides a useful conceptual framework. Although evidence specific to this setting remains limited and largely descriptive, acute pyelonephritis could plausibly contribute to each component of the triad through interrelated local and systemic pathways. Figure 2 schematically summarizes these proposed mechanisms.

First, venous stasis may reflect both baseline anatomic and hemodynamic features of the ovarian vein and superimposed locoregional inflammation. The ovarian vein runs retroperitoneally alongside the ureter [13], displays asymmetric drainage, and exhibits valvular features, including valvular incompetence and reported absence of valves [4], which may predispose to low-flow states and favor stasis. In addition, periureteral inflammation and edema related to pyelonephritis and ureteritis may further impair venous outflow through local mass effect and the close retroperitoneal contiguity between the ureter and ovarian vein [4,13]. The present case and previous reports support the biologic plausibility of such a mechanism [5-6,14].

Second, local endothelial injury may be promoted by perivenous inflammation related to pyelonephritis and ureteritis. Several case reports, including the present case, suggest that this process may result from local inflammatory spread both by contiguity and via ureteral venous drainage pathways involving the ovarian vein [6,13-14]. At the systemic level, pyelonephritis-associated inflammation may contribute to endothelial activation, barrier dysfunction, and the acquisition of a pro-adhesive, procoagulant phenotype [15-17]. Third, hypercoagulability may arise locally from perivenous inflammation, which promotes a prothrombotic perivascular milieu and amplifies coagulation activation in a vein already predisposed to low-flow conditions [6]. Systemically, it may reflect infection-associated prothrombotic signaling, as infections can engage procoagulant pathways along a continuum often discussed within the broader spectrum of thrombo-inflammation [15-17].

Epidemiologic data further support an association between symptomatic urinary tract infection (UTI) and increased VTE risk, suggesting that UTI may lower the threshold for thrombosis [18]. In this patient, combined estrogen-progestin contraception and smoking likely contributed to an additional prothrombotic background and may have amplified the infection-related contribution to Virchow’s triad [4,15-17]. Thus, OVT arising in the setting of pyelonephritis may reflect combined contributions across all three components of Virchow’s triad. However, this remains a mechanistic, hypothesis-generating interpretation based primarily on biologic plausibility and descriptive observations. Accordingly, pyelonephritis is best regarded as an associated clinical context and a possible precipitating factor rather than an established cause.

Outside the postpartum period, OVT is an uncommon and often overlooked cause of acute abdominal or pelvic pain in the ED. Its presentation is nonspecific and may overlap with more common gastrointestinal, urinary, and gynecologic emergencies [1,4]. OVT should be considered when predisposing factors are present and reconsidered when the clinical course is atypical, particularly if expected improvement does not occur despite appropriate initial management. In the acute setting, contrast-enhanced abdominopelvic CT is the most commonly used diagnostic modality, as it can identify OVT, assess extension to the renal vein and/or inferior vena cava, and simultaneously evaluate alternative diagnoses [1,19-20]. In such situations, targeted radiologic reassessment, including systematic evaluation of the abdominopelvic veins on the initial CT examination or repeat imaging when clinically justified, may help reduce missed diagnoses of OVT [1]. In our case, the initial CT was interpreted as showing acute pyelonephritis on the preliminary on-call CT report, and OVT was identified only on final review of the same examination by an attending radiologist on day three, illustrating this diagnostic challenge in emergency practice.

A second limitation is that recanalization could not be radiologically documented. Planned follow-up CT was canceled after early pregnancy was identified, and the ultrasound was inconclusive for reliable assessment of ovarian vein patency [1,19-20]. Nevertheless, the favorable clinical course, together with the high recanalization rate reported with anticoagulation in published series (89.4%) [21], made persistent thrombosis less likely. For this reason, no additional cross-sectional imaging was pursued during pregnancy. Pulmonary embolism complicates OVT in approximately 6.5% of cases [1], whereas concomitant pulmonary embolism has been reported in 49-72% of lower-extremity DVT cases when systematically sought [22]. Accordingly, on day three, the YEARS algorithm [7] was negative, and CTPA was not performed because its result was unlikely to alter immediate management.

Beyond imaging itself, this case highlights the value of final review of preliminary on-call CT interpretations by an attending radiologist, particularly during off-hours, when a more common diagnosis may overshadow a rarer entity such as OVT [1]. In on-call cohorts, discrepancies between preliminary and final abdominal CT reports occur in approximately 17% of examinations, with 7% considered major or clinically significant [23]. Such discrepancies and missed diagnoses may be promoted by incomplete or inconsistent referral information, potentially biasing interpretation [24], on-call constraints such as workload, interruptions, and fatigue, which underscore the need for double-reading pathways [25], and limited familiarity with abdominopelvic vascular entities such as OVT, which may be overlooked once an initial working diagnosis has been established [1,23].

In the absence of randomized controlled trials and a dedicated standard for non-obstetric OVT, management relies on extrapolation from conventional VTE practice and observational data [21]. Available guidance on thrombosis at unusual sites addresses OVT mainly in the postpartum setting and supports individualized treatment decisions, particularly because pivotal trials of direct oral anticoagulants (DOACs) excluded patients with unusual-site VTE [26-27]. In our case, a three-month DOAC-based regimen was therefore a reasonable choice [21,28-29]. OVT-specific data remain limited but are reassuring, with DOACs, particularly rivaroxaban, appearing effective and associated with a low rate of major bleeding (1.27%) [21,29]. Finally, pyelonephritis may precipitate OVT but does not preclude its diagnosis; when infection is present, antibiotic therapy remains a cornerstone of management [3,21].

## Conclusions

Non-obstetric OVT is a rare but clinically important diagnostic consideration in women presenting to the ED with acute abdominal or pelvic pain and fever. Because its presentation is nonspecific, it may mimic more common urinary, gastrointestinal, and gynecologic emergencies and can be missed on the initial interpretation of contrast-enhanced abdominopelvic CT. Clinical reassessment and final CT review by an attending radiologist may therefore be crucial when symptoms fail to improve despite appropriate initial management. Awareness is particularly important when prothrombotic risk factors are present, such as estrogen exposure, smoking, recent surgery, malignancy, or urogenital/intra-abdominal infection. Early recognition can prompt timely therapeutic anticoagulation and reduce the risk of complications. This report underscores the value of including OVT in the differential diagnosis and of systematically reassessing the ovarian and other abdominopelvic veins on CT in women with acute abdominal or pelvic pain.
